# Floor-hugging Intervention: A Perspective on Floor Exposure and After-Fall Contingency Intervention

**DOI:** 10.1177/27536351241271548

**Published:** 2024-08-22

**Authors:** Shashank Ghai, Ishan Ghai

**Affiliations:** 1 Department of Political, Historical, Religious and Cultural Studies, Karlstad University, Karlstad, Sweden; 2 Centre for Societal Risk Research, Karlstad University, Karlstad, Sweden; 3 School of Life Sciences, Jacobs University Bremen, Bremen, Germany

**Keywords:** Fear of falling, fall contingency, floor-rise strategy, falls injuries

## Abstract

The fear of falling is a pressing public health issue, yet current interventions often fall short in addressing it effectively. As a result, there is a need for innovative interventions that go beyond symptom relief to address the underlying causes. From this standpoint, we propose that limited exposure to floors and a lack of post-fall contingencies may contribute to the uncertainty that amplifies the fear of falling, particularly in fall prone populations. We explore the theoretical underpinnings of this hypothesis and propose a framework based on the Uncertainty and Anticipation model to elucidate potential connections. Building upon this, we introduce the Floor-hugging intervention—a two-part strategy designed to confront these challenges. Firstly, we propose gradual exposure to different floor scenarios through guided imagery to diminish fear by familiarizing individuals with such situations. Secondly, we advocate for the adoption of evidence-based ways to get up from the floor for developing after fall contingencies. We delve into the theoretical framework supporting our approach and its potential to reduce the fear of falling while improving physical, social, and psychological well-being. Additionally, we outline prospective outcome measures to comprehensively assess the impact of the intervention across biopsychosocial domains. This perspective aims to stimulate discussion on the potential role of floor exposure and post-fall strategies in reducing the fear of falling, while also advocating for innovative interventions to empower and protect fall-prone populations.

## Background

Falls represent a significant public health concern worldwide.^1^ Existing literature has widely underscored falls as a major contributor to physical morbidity and mortality worldwide.^2^ While predominantly observed in older adults, falls are not uncommon in other younger population groups.^3^ In addition to physical harm, people who experience falls,^4^ as well as those who don’t,^5^ often exhibit heightened levels of anxiety, particularly in the form of fear of falling.^6^ Fear of falling is characterized by a lack of confidence in one’s balance abilities to prevent falls during daily activities.^7^ This fear can range from a healthy caution that causes individuals to avoid environmental hazards such as icy roads or wet surfaces, to a disabling fear that prevents individuals from engaging in activities that they are capable of performing.^7^

**Figure 1.**
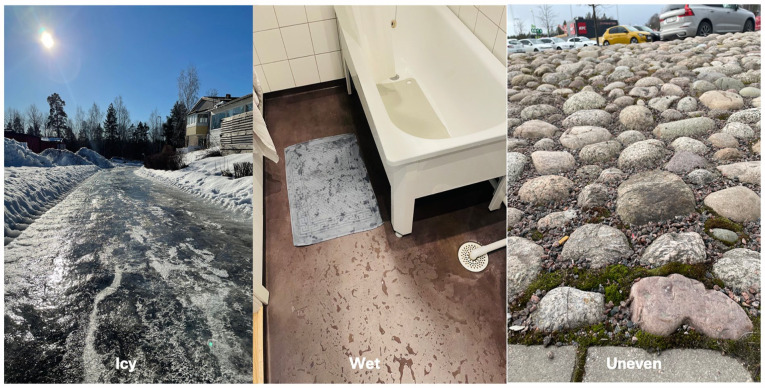
High risk floors.

Various factors, spanning biological, cognitive, psychological, social, and environmental domains, have been identified in the literature as contributing to the prevalence of fear of falling.^8^ For instance, among older adults, age-related declines in musculoskeletal strength and sensory perception are notable factors predisposing individuals to an increased risk of accidental falls and injuries, with fear of falling emerging as a consequential outcome.^9^ In addition, cognitive decline, particularly in executive functions, reduces the ability to accurately perceive and respond to environmental cues, thereby exacerbating fear of falling.^10^ Besides, environmental factors also play a significant role in increasing both falls and associated fear.^11^ As an example, wet, uneven, inclined, dark or slippery surfaces (Figure 1), clutter, and lack of accessibility features all impede mobility and heighten the risk of falls, adding to the anxiety of navigating one’s environment.^11^ Consequently, these factors contribute to deteriorating psychological well-being, marked by heightened levels of anxiety, depression, and embarrassment leading to unintentional self-imposed limitations on mobility.^12,13^ This activity avoidance behavior in turn leads to poor physical outcomes, increased social isolation, and loneliness perpetuating a cycle of decline in both biological factors and overall quality of life.^14^ Fear of not being able to get up on one’s own is another important factor that exacerbates the fear of falling.^14^ Although existing interventions have been designed to address the fear of falling in older adults, their effectiveness typically ranges from small to moderate effect sizes.^15,16^ Moreover, questions persist regarding the long-term effectiveness of these interventions.^17^ The limited effectiveness of existing interventions may be due to their failure to address the risk of accidental falls, the risk of injury during falls and embarrassment as specific components, suggesting that different multifaceted approaches may be needed to address fear of falling.

An important factor that may explain this lack of sustained improvements in current interventions may be that the literature has largely overlooked the role of the design of our environment, particularly in relation to the individual’s relationship with the floor itself.^14^ Research suggests that harder, uneven or slippery floors can trigger fear of falling, even when they are actually safe.^18^ This suggests that fear of falling may not be driven by the actual risk of falling, but by the sensory cues that indicate potential danger. We believe that exploring this concept may provide greater insight into how we can manipulate this relationship to reduce fear of falling.

In modern industrialized societies, daily activities rarely involve prolonged contact with the floor.^19^ A recent study by Fajzel et al^20^ found that globally, most waking hours are spent on passive, social, and interactive activities (approximately 31%), followed by activities driven by external factors (22%), and those motivated by organizational outcomes (14%), all of which do not involve direct floor contact. To illustrate, a typical workday for the average American worker may involve up to 13 hours of passive sitting on a chair, followed by 3 hours of physical activity, and 8 hours of sleep.^21^ This lack of exposure to the floor in contemporary urbanized societies, partly due to the prevalence of chairs and societal norms that discourage sitting on the floor, may explain why our daily activities rarely involve direct interaction with the floor beyond basic postures such as standing, sitting on a chair.^19^ This limited engagement with the floor means that activities like sitting, lying down, or even kneeling on the floor are not as common in our daily lives as they may have been in previous eras or in cultures where floor-based activities such as cross-legged sitting or deep squat sitting are more prevalent.^19,22^

We hypothesize that this lack of exposure could lead to a disconnection between individuals and their awareness of the floor beneath them. Besides, the lack of exposure to the floor could be more than just physical contact; it could also involve mental and sensory familiarity with the floor. For example, without regular contact with the floor in various positions, individuals may feel uncertain or unfamiliar with it. This uncertainty is similar to someone encountering utensils such as fork and knife they’ve never used before, or adapting to driving on the opposite side of the road in a new country. Lack of familiarity with either the tools or the driving norms can intensify feelings of uncertainty and potentially lead to anxiety. Similarly, we theorize that the limited exposure to the floor in modern culture has contributed to a sense of uncertainty, fostering a negative bias by viewing the floor as unfamiliar or even a foreign entity. In the context of fear of falling, this perception of uncertainty has inadvertently increased fear of falling because the floor is seen as a potential threat rather than a stable support. Conversely, in Eastern cultures such as India, where sitting on the floor is deeply rooted in tradition, postures such as sitting cross-legged, kneeling, and squatting are seen not only as comfortable but also as desirable rehabilitative goals.^22^ Samant et al^23^ found that people in India who retained the ability to sit and rise from the floor reported lower levels of fear of falling than to those who did not. This may potentially reflect their confidence in being able to get up from the floor themselves, as they practice this in their daily lives.

Furthermore, a lack of familiarity with floor might also play a role in hindering an individual’s ability to respond effectively to falls, often leaving them unsure of what steps to take next. Cox and Williams^24^ suggested that the fear and anxiety associated with falling may also arise from concerns about being unable to recover from a fall. This lack of preparedness for post-fall situations may be due to an individual’s reluctance to learn these essential coping skills,^25^ or perhaps the reluctance of physiotherapists to teach them.^26^ The fear-avoidance model,^8^ and the comfort-stretch-panic model,^27^ provide insight into this hesitancy to learn, suggesting that individuals prefer to operate in anxiety-neutral conditions and may go to great lengths to avoid stressful and uncertain situations. In addition, biological processes may interfere the the encoding of safety information and exacerbate cognitive-motor interference, hindering learning in uncertain circumstances.^27^ In our context, uncertainty about the floor due to lack of exposure may exacerbate anxiety and inhibit the learning of floor-rising strategies, thus further intensifying the fear of falling.

### Detailed framework of our uncertainty hypothesis

**Figure 2.**
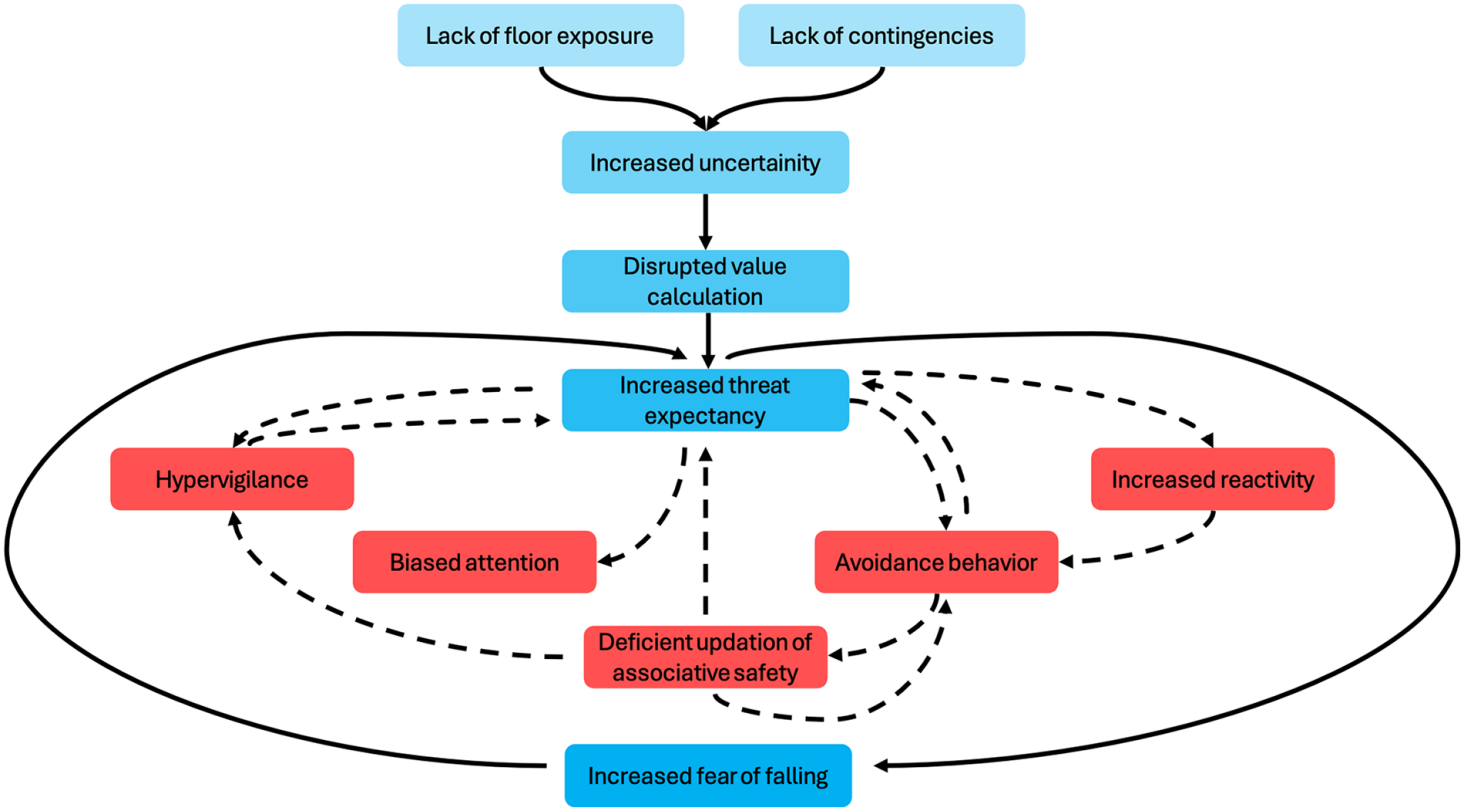
Adapted model portraying amplified fear of falling due to limited floor exposure, rooted in the Uncertainty and Anticipation Model of Anxiety.^28^ The dotted lines and red boxes denote five internal processes escalating threat anticipation from uncertainty.

Our hypothesis that the fear of falling is being fueled by lack of floor exposure and the absence of post-fall contingencies is consistent with the Uncertainty and Anticipation (UAMA) model. The UAMA model (Figure 2) proposes that neural networks exhibit maladaptive functioning in response to uncertainty about threats, leading to maladaptive cognitive, behavioral, and affective responses that ultimately result in heightened anxiety.^28^ The model identifies 5 main processes that explain adaptive anticipatory responses to future threat uncertainty, resulting in maladaptive responses, such as pathological anxiety, which in this case could manifest as a fear of falling.

Here, a lack of exposure to the floor and a lack of knowledge about how to get back up after a fall could increase an individual’s uncertainty and anticipatory distress, thereby inflating their estimates of the threat costs of uncertain negative events, such as a fall.^29^ Moreover, there is evidence that threat expectancy adjustment can be impaired by aversive predictions resulting from anticipatory distress when a predicted negative event, such as a fall, does not occur.^30^ The literature suggests that this impairment in threat expectancy adjustment may result from maladaptive activity in the ventral striatum, anterior insula and rostral cingulate cortex.^28,31^ However, this does not mean that some people’s confidence cannot increase appropriately over time without a fall. Secondly, uncertainty about the floor and the absence of contingencies may also induce hypervigilant behavior in individuals, leading to an attentional bias and an increased tendency to interpret ambiguous stimuli as threatening. This concept finds support in the learning model proposed by Pearce and Hall,^32^ which suggests that unpredictable environmental cues can lead to an inefficient allocation of attentional resources, resulting in hypervigilance and impaired stimulus-outcome associations, even in objectively predictable environments. In our scenario, individuals may exhibit hypervigilance when navigating high-stress environments, and the inefficient allocation of attentional resources may contribute to cognitive-motor interference, ultimately increasing the risk of falling.^33^

Thirdly, uncertainty and lack of contingency may also impede the discriminative analysis of environmental cues in individuals lacking floor exposure to identify safety signals, potentially leading to heightened reactivity. For example, an individual experiencing high levels of uncertainty may have difficulty identifying safety signals in stressful environments. This difficulty could lead them to overreact to minor changes such as slight variations in texture or lighting, thereby increasing the risk of a fall. Fourthly, the caused by a lack of floor exposure might foster avoidance behavior in people with a fear of falling.^8^ Avoidance behavior may increase threat expectancies through unintentional negative reinforcement, leading to the development of false beliefs. This behavior may result from the intolerable state created by negative anticipatory emotions due to uncertain conditions. For example, an individual may avoid certain activities or environments due to uncertainty about their safety. This avoidance behavior inadvertently reinforces their fear of falling, leading to false beliefs about their abilities and perpetuating a cycle of fear and immobility. Fifthly, conditions of uncertainty may induce motor inhibition to carry out risk assessment and resolve conflicts between competing options or motivating factors. For people with a heightened fear of falling, uncertainty in a high-stress environment could lead to increased motor inhibition, manifested as hesitation or freezing, which could increase the likelihood of a fall.^34^

## Methodology

To gain a comprehensive understanding of the existing literature on floor exposure and floor-rise interventions, we conducted a non-systematic literature search across multiple databases to ensure a wide coverage of relevant studies. The databases searched included PubMed, Scopus, Web of Science, PEDro, and CINAHL. The search was conducted using a combination of the following key words: “falls,” “accidental falls,” “floor exposure,” “exposure therapy,” “fear of falling,” “after-fall strategies,” “fall contingencies,” “floor-rise strategies,” “backward chaining,” “forward chaining,” “lie-to-stand,” “long lie,” and “long lie episode.”

### Floor hugging intervention and its theoretical underpinnings

The UAMA model not only highlights how limited exposure to the floor can exacerbate uncertainty and promote the development of the fear of falling but also provides opportunities to develop targeted interventions to mitigate this uncertainty and effectively manage fear of falling. For instance, the innovative “floor hugging intervention” (Floor-HI) proposed in this perspective is designed with this in mind and aims to promote safe exploration and proactive interaction with the environment to mitigate the negative effects of uncertainty on fall-related outcomes. The Floor-HI program could provide a potential solution by encouraging active engagement with uncertain floor surfaces, thereby potentially addressing the fear of falling and its associated health risks.

The Floor-HI program consists of 2 main components, one of which is dedicated to acclimatization to the floor, while the other focuses on establishing protocols for getting up from the floor after a fall. In the first phase, the Floor-HI program involves a process of acclimatization and exposure to the floor, aimed at mitigating the apprehension or uncertainty that individuals may feel about floor-related activities, and thereby potentially reducing the fear of falling. The theoretical foundation of this intervention draws from the emotional processing theory, which underscores the need for confrontation with aversive stimuli in interventions targeting fear or anxiety disorders.^35^ According to this theory, such confrontations activate cognitive fear structures in a safe environment, facilitating the possibility of reshaping, competing with, or updating the original associations between threat and safety.^36^ In the proposed intervention, we intend to implement the Floor-HI program by asking an individual to lie on the floor in different positions and in different environments and then asking them to imagine that they have fallen. This visualization will be guided, with participants imagining the fall but understanding that it is separate from an actual fall, ensuring that they feel safe. Additionally, the guided imagery will incorporate relaxation techniques, such as deep breathing exercises and soothing meditation music, to help alleviate any heightened anxiety.^37^

By following this procedure, and during repeated exposure to the floor while imagining a fall, this could potentially activate the associative fear networks, albeit in a safe, controlled environment, thereby allowing systemic desensitization to the uncertainty for a number of reasons, including cognitive restructuring, increased self-efficacy, counter-conditioning, habituation, excitation, and dissonance.^38^ For example, cognitive restructuring can occur when participants challenge their belief that falling is always harmful. Through repeated exposure to the floor and repeated practice getting up, participants can build confidence by improving their ability to get back up from the floor. Additionally, counter-conditioning could occur as participants associate the floor with feelings of safety and relaxation rather than fear and anxiety. Similarly, over time, participants could become accustomed to the fear of falling, experiencing reduced anxiety with each session. Initially experiencing excitation as they confront their fear, participants could gradually learn to manage their anxiety and discomfort, leading to reduced fear and increased comfort with different floor environments. Throughout this process, participants might also experience cognitive dissonance as they reconcile their fear of falling with the evidence of safety provided by the intervention, ultimately leading to a reduction in fear and an increased sense of confidence and safety.

**Figure 3.**
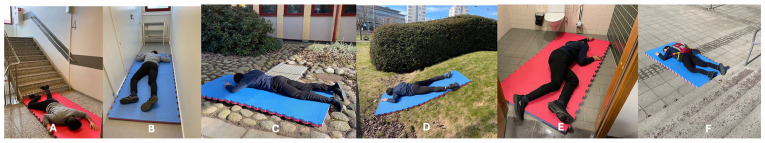
Different positions and environments demonstrating the variability in the floor-hugging intervention: (A) stairs indoor, (B) narrow pathway with steel steps, (C) uneven surface, (D) inclined slope, (E) wet bathroom, and (F) stairs outdoor with bag.

Moreover, given the perspective of the inhibitory learning model, which suggests that fear associations persist alongside inhibitory (“safety-based”) associations during exposure therapy, our goal is to cultivate novel inhibitory safety-based associations that remain accessible across various contexts and over time.^37^ To achieve sustained improvements in line this model, we propose to introduce variability into the learning process to robustly update safety associative learning. This variability can be introduced by delivering the Floor-HI program in different environmental contexts with high ecological validity, such as bathrooms, staircases, uneven surfaces, narrow passageways, and dimly lit areas, where falls are common.^39^ Additionally, participants could practice assuming different positions that they might envision falling and lying, not just conventional supine, side-lying, or prone positions, but also semi-prone/supine positions. Variability can also be incorporated by adjusting the level of base support; for example, initial training could commence with a conventional 40 mm sports mat to ensure comfort and reduce pressure on bony prominences. However, as training progresses, the thickness of the mat can be gradually reduced to progressively improve tactile feedback with the floor, maximizing its ecological salience. Similarly, the integration of accessories such as backpacks or grocery bags during floor acclimatization, as well as experimentation with various types of footwear, including high heels, could enhance the formation of safety-related associations applicable across different scenarios. By increasing the ecological salience during the Floor-HI sessions and encouraging participants to practice multiple positions reflecting potential fall scenarios, alongside real-world applicability throughout training, we hypothesize that numerous safety-based inhibitory associations will be formed. As a result, these associations may remain accessible across in different contexts and persist over time (Figure 3).

In the existing literature, although limited studies have investigated the efficacy of exposure-based therapies in mitigating fear of falling 40 to 43, these interventions are still at an early stage of development. Existing exposure therapies for fear of falling are primarily limited by their scope and approach. For example, the Activity, Balance, Learning, and Exposure (ABLE) intervention used exposure-based cognitive behavioral techniques, such as identifying negative thoughts and promoting realistic alternatives.^40^ Similarly, the Back to My Feet intervention introduced a novel form of exposure therapy that included both imaginal and in vivo forms of exposure. Here, patients were involved in narrating their experiences of falling, while therapists facilitated a gradual confrontation with the avoided activities.^41^ Despite the novelty of these interventions, it’s worth noting that none of the current exposure-based interventions directly address the act of falling itself. Besides, existing exposure therapies are primarily tailored for people with a history of falls. Their reliance on recounting past falls and revisiting fall-prone environments limits their utility, particularly as a preventive measure for people who are at risk of falling have not yet experienced a fall.^42^

**Figure 4.**
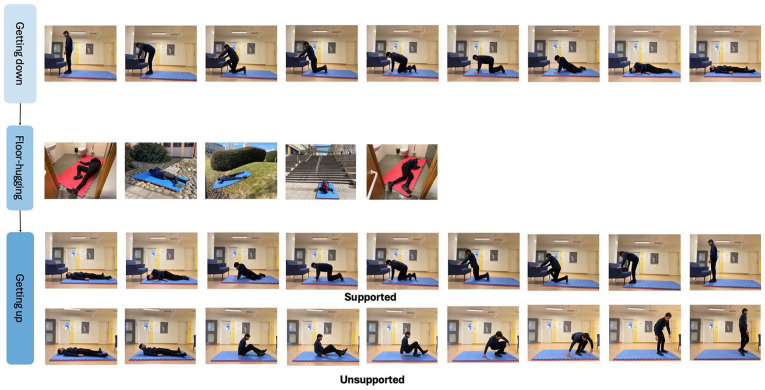
Schematics of the intervention.

The exposure component of the Floor-HI program addresses the gaps in existing exposure interventions by providing direct exposure to the fear induced by the act of falling. Specifically, the Floor-HI program emphasizes global exposure to the fear of falling in a variety of settings and can also be used as a prophylactic intervention in individuals without no history of falls. Furthermore, unlike existing exposure-based therapies, which primarily incorporate exercises,^40,43^ the second part of the Floor-HI intervention is dedicated to developing ways to get back up from the floor. This approach aims to empower individuals by providing them with practical skills to effectively recover from falls. By focusing on the development of post-fall contingency plans, the intervention aims to increase individual self-efficacy in managing falls and address the issue of prolonged periods of lying on the floor after a fall, commonly known as “long lie” episodes.^44^ The prolonged periods of immobility after a fall not only worsen morbidity and mortality outcomes but also have a detrimental effect on psychological well-being, leading to heightened feelings of fear and helplessness, a concern that is increasingly recognized worldwide.^44,45^ Therefore, the implementation of post-fall contingencies aims to improve safety and control of anticipatory responses, thereby reducing the impact of future falls, even when complete prevention is not possible. Participants in the Floor-HI program will be introduced to evidence-based, structured, floor rising strategies aimed at enhancing their ability to effectively rise from the floor.^46-48^ These strategies are structured within the program to first familiarize individuals with lying on the floor, allowing them to acclimatize to the floor, before progressing to practicing various approaches to rising from different positions (see Figure 4).

### Structure of the intervention

The intervention is structured to systematically address individual uncertainty about the floor and to develop post-fall contingencies. Central to this structured approach is the use of evidence-based floor-rise strategies, that guide participants through the process of both assuming a specific position on the floor and regaining an upright posture. Based on the existing literature, which recommends a training dosage of 20 to 30 minutes each for the benefits of floor-rise strategies such as the backward chaining method,^46^ and exposure-based therapies,^40,41^ we hypothesize that the Floor-HI program would require a training dosage of 40 to 60 minutes per session. This duration would allow for an equal distribution of floor exposure and floor rise training. Sessions could be performed 3 times a week for a minimum of 8 weeks. Specifically, the intervention would being with participants using the backward chaining method to assume a predetermined position on the floor, simulating a fall scenario, followed by a supported or unsupported floor rise strategy to regain an upright stance.

**1. Positioning on the floor:** The first step would be to use the backward chaining method Leonhardt et al^46^ which involves using assistive furniture to transition from stride standing to half kneeling, then to high kneeling, prone kneeling, half sitting, and finally to side, prone, or supine lying, facilitating the acclimatization phase to the floor.
**2. Floor-acclimatization**: During the floor acclimatization process, participants will be guided to lie down on the floor in various positions, including supine, prone, side lying, and semi-prone/supine positions. Initially, they will be asked to observe the environment as if they have fallen, taking in the surroundings. Subsequently, with their eyes closed, participants will engage in guided imagery exercises, where they will imagine themselves experiencing a fall. Throughout this process, participants will be instructed to perform deep breathing relaxation exercises to alleviate fall-associated anxiety. Additionally, they will receive structured guidance during the imagery exercise, to focus on positive outcomes to counteract any negative emotions and create dissonance with the fall event.
**3. Getting back up from the floor**: The third step will involve teaching strategies for getting back up from the floor tailored according to individual balance capabilities. For instance, participants with poor balance abilities will receive training in getting back up from the floor using the forward chaining method, which is essentially a reverse of the backward chaining method. This method incorporates transitions from supine to side-lying, to half-sitting, to prone kneeling, to high kneeling, to half-kneeling, and finally to stride standing using assistive furniture. For people with better balance, such as young or middle-aged individuals, a comprehensive without assistive furniture developed by Schwickert et al^48^ could be used. This method consists of 7 core components: lying, initiating, positioning, supporting, elevation, stabilization, and finally transitioning to either a standing or stride posture. The method by Schwickert et al,^48^ although challenging, is intended to provide a wide range of evidence-based options for getting back up from the floor. Overall, this tailored approach will ensure that individuals with varying levels of mobility and balance can benefit, thereby promoting confidence and reducing the fear of falling in diverse populations.

In terms of floor rising strategies, both the backward and forward chaining methods for getting down and rising from the floor, respectively, adhere to a structured training approach outlined by Adams and Tyson.^49^ This method has been shown to be effective in evidence-based outcomes and has been qualitatively reported to be more acceptable than conventional unstructured floor rising strategies.^46,47^ Additionally, the comprehensive model developed by Schwickert et al^48^ has been shown to improve functional performance when rising from the floor without assistance. In the Floor-HI intervention, the aim of moving from supported to unsupported floor rising strategies is to address the gap in existing governmental guidelines on floor rising,^50,51^ which often advocate the use of furniture for getting back up after a fall without considering environments where such support may not be available, such as outdoors. Additionally, to ensure the personalization of the intervention, the procedure for getting up and down on the floor will be tailored to individual needs. For example, foam wedges may be used for initial half kneeling, and pillows may be used for support during half sitting for participants who lack flexibility or strength.^47^ Furthermore, during the Floor-HI program, aspects of the intervention could be adapted based on individual capacity to rise from the floor. Emphasis could also be placed on maintaining the quadruped position, particularly for individuals using support to rise from the floor. The aim is to develop endurance in the quadruped position to enable individuals to access support furniture in the event of a fall if they do not have sufficient strength to rise independently. We also hypothesize that the weight-bearing imposed on the joints will cause inter-joint approximation, such as during the quadruped position and the supporting and elevation components where the individual rises unsupported from supine to standing position with arms extended behind them. These positions could be beneficial in developing joint stability and strength.

Furthermore, we hypothesize that repeated training of floor rising strategies could strengthen the upper and lower extremities and the core, thereby reducing the risk of accidental falls and injury, fear of falling, while improving floor rising ability and balance outcomes.^52^ By engaging different muscle groups when practising these strategies, they not only promote muscle strengthening but also challenge joint stability and coordination. This includes maintaining weight-bearing positions such as the quadruped position, which can improve neuromuscular control, stability, and proprioception, crucial for balance maintenance.^52^ Similarly, for people wishing to maintain existing abilities, regular practise in getting up from the floor, perhaps as part of a daily routine, could reduce the uncertainty, embarrassment and fear of falling by increasing confidence and self-efficacy in their ability to get back up after falling.

While the Floor-HI program aims to reduce fear of falling and improve acclimatization to the floor, it is important to consider potential adverse effects as well. For instance, participants may experience initial discomfort or anxiety when exposed to the floor, which could temporarily increase their fear before desensitization occurs. The physical demands of the intervention, including the transition from floor to standing, may pose a risk of musculoskeletal strain or injury, particularly in individuals with pre-existing conditions or limited physical capacity. Careful screening, tailored modifications and close monitoring during sessions are essential to minimize these risks and ensure participant safety.

### Assessment of performance

**Figure 5.**
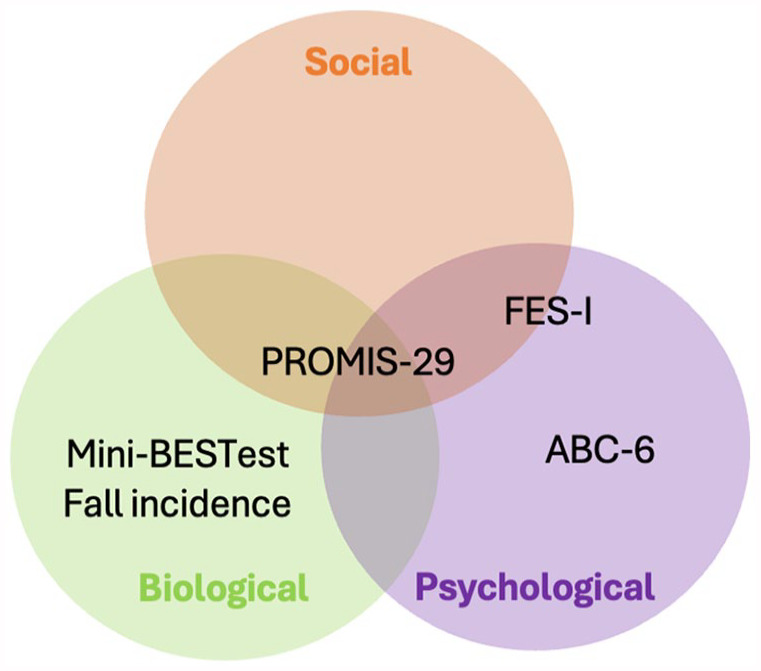
Outcome measures for assessments of floor-HI assessment based on biopsychosocial model.

The Floor-HI program has been specifically designed in line with the biopsychosocial model of the International Classification of Functioning, Disability, and Health,^53^ which recognizes the complex interaction between biological, psychological, and social factors in promoting individual well-being. The primary aim of the intervention is to reduce the uncertainty associated with floor exposure and post-fall contingencies, thereby mitigating psychological distress, particularly heightened anxiety and fear of falling. Additionally, the program aims to reduce the functional mobility constraints resulting from fear of falling, which often lead to social limitations and isolation.^6^ It is anticipated that by reducing fear of falling and improving balance and mobility outcomes, there will be a positive impact on the social wellbeing of participants. Furthermore, improvements in physical outcomes, such as balance and mobility, are expected through reduced uncertainty and repeated practice of floor rising strategies, as evidenced in the existing literature. In order to comprehensively assess the impact of the Floor-HI intervention on mobility outcomes, the following assessment scales have been proposed to capture its broader effects across various domains (Figure 5):

1. Fear of falling: Fear of falling could be assessed using the 16-item falls efficacy scale-international scale. The scale assesses fear of falling during activities of daily living, which are also sensitive for active adults and also assesses the social dimension of the fear of falling and has been shown to have excellent measurement properties even in a cross-cultural context.^54^
2. Activity and balance confidence: The assessment of activity and balance confidence could be performed by the 6-item short version of the Activities Specific Balance Confidence scale. The scale has been reported to be a reliable and valid tool for assessing activity and balance confidence in community-dwelling older adults.^55^ The scale includes important activities of daily living such as, standing on tiptoe to reach for something, standing on a chair to reach for an object, navigating through crowded spaces, walking on escalators, and traversing icy sidewalks, which are prominent in day-to-day life settings and augment fall-related uncertainty.
3. Sitting rising test: The untimed test could be used to assess individual ability to rise from the floor. The 5-point test assesses independence (ie, lack of support) and stability (ie, clinician-reported) in the ability to rise from the floor and has been shown to be responsive, reliable, and valid.^56^
4. Static and dynamic stability: The Mini-BESTest could be used to assess the influence of the Floor-HI program on static and dynamic balance outcomes. The assessment covers a wide range aspects of balance control. It assesses anticipatory postural adjustments, reactive responses, sensory integration, dynamic gait, stability limits, trunk stability, dual task performance, and sitting balance. The assessment has been reported to be highly sensitive, reliable, and valid for assessing balance impairment in older adults.^57^
5. Patient-Reported Outcomes Measurement Information System (PROMIS) 29-item could be used to assess the overall wellbeing of the participants undergoing the Floor-HI program. The survey evaluates 7 health domains, including physical function, fatigue, depressive symptoms, anxiety, pain interference, sleep disturbance, and participation in social roles and activities. Each domain is rated on a five-point Likert scale, and the scores are converted into standardized T-scores. PROMIS-29 has been reported to be a highly responsive, reliable and valid instrument for assessing health-related quality of life.^58^
6. Self-reported fall incidence could also be recorded using a fall diary, which records information about the location of the fall, the perceived cause of the fall, the activity associated with the fall, the landing of the fall and its consequences.^59^

We selected these assessment scales based on their alignment with the aims of our intervention and the biopsychosocial model. These scales were specifically chosen for their robust measurement properties, as supported by previous research, which further justified their selection for evaluation of the Floor-HI program.

### Safety, contraindications and limitations

The Floor-HI program should be implemented with safety in mind by ensuring that it is carried out under the supervision of trained healthcare professionals, such as physiotherapists or occupational therapists, who are knowledgeable about fall prevention and rehabilitation. These professionals can guide participants in the correct and safe implementation of the floor acclimatization and rising strategies. In addition, before the intervention begins, each participant must undergo a thorough assessment to evaluate their physical abilities, medical history and fall risk factors to ensure that the program is tailored to their specific needs and that the floor rising strategies are assigned according to individual health capabilities to prevent discomfort, fatigue or injury. Sessions also need to take place in a safe, controlled environment, such as a living lab,^60^ that mimics real life settings but is free of hazards such as sharp objects, slippery surfaces or obstacles, with appropriate padding or mats to cushion the floor and minimize the risk of injury. In addition, the program must be progressive, allowing participants to start at their comfort level and slowly progress to more challenging positions as their confidence and skills improve, and there must be regular communication between participants and healthcare professionals to address concerns and adjust the program as needed.

Similarly, the Floor HI program should be carefully considered for individuals with certain contraindications. Individuals with acute medical conditions such as recent fractures, injuries or surgery may not be suitable candidates until they have fully recovered and received clearance from their healthcare provider. People with severe cognitive impairment or dementia may have difficulty understanding and following the instructions for the Floor-HI program, limiting its effectiveness for them. In addition, people with severe balance or mobility problems may require additional support or modifications to participate safely, and in some cases alternative interventions may be more appropriate to meet their needs.

Although this perspective offers a novel way of dealing with fear of falling, it has some limitations. First, the development of the proposed Floor-HI program relies heavily on psychological theories such as emotional processing theory and the inhibitory learning model. While these theories are well established and have guided the development of effective exposure-based interventions for the management of anxiety and fear-related disorders, an empirical evaluation of the proposed intervention is required to determine its effectiveness. Secondly, although the corresponding author of this proposed intervention is a physiotherapist with experience in geriatric care, we did not engage in discussions with physiotherapists who manage post-fall contingencies on a daily basis. This may limit the practical insights into the application of our proposed intervention. Thirdly, as this was a perspective article proposing a novel intervention, we did not carry out a formal strength of evidence assessment of the studies. The main reason for this decision was to maintain a broad and inclusive review, capturing the different approaches and emerging trends in the field of floor exposure and floor rise interventions. Given the relatively early and exploratory nature of this area of research, many innovative interventions have been presented as case reports,^40,41,61^ that may not fit neatly into traditional evidence hierarchies. By not restricting our review to high-level evidence, we aimed to provide a more comprehensive overview of the current research landscape and highlight how the novel interventions presented in this perspective might address gaps in the current state of the literature. We therefore recommend that future studies take a systematic approach to assessing the state of the literature, using an appropriate strength of evidence analysis. Fourth, as the suggested training duration for the Floor-HI program is based on the findings of existing literature, we believe that there may be inherent limitations in the studies, such as individual variability and intervention heterogeneity, which could affect the effectiveness of the Floor-HI program. Therefore, future studies should use rigorous dose-response analysis to determine the optimal dose and improve our understanding of the effectiveness of the program.

## Summary

In summary, falls are a pervasive public health problem that affects physical, psychological, and social wellbeing across various age groups. Fear of falling, exacerbated by uncertainty about floor exposure and post-fall contingencies, amplifies anxiety and hampers mobility and quality of life. The Floor-HI program, based on evidence-based strategies and theoretical frameworks, offers a promising way to address these challenges. By systematically addressing uncertainty through floor acclimatization and post-fall contingency training, Floor-HI program aims to reduce fear of falling, enhance balance and mobility outcomes, and ultimately improve overall well-being. Comprehensive evaluation criteria, based on the biopsychosocial model, can be used to rigorously assess the effectiveness of the Floor-HI program and provide insights into its potential to mitigate the multifaceted impacts of falls and fear of falling. It’s also important to emphasize that the aim of this perspective is not to criticize existing interventions, but to stimulate a discussion in the community with a fresh perspective. By encouraging dialog and exploring of innovative approaches, we aim to collectively advance our understanding and management of the complex issue of fear of falling.
